# Combination Use of Tegafur and Apatinib as First-Line Therapy in Treatment of Advanced Gastric Cancer: A Single-Blinded Randomized Study

**DOI:** 10.1155/2020/3232950

**Published:** 2020-04-06

**Authors:** Chaofeng Li, Tao Tang, Wenyue Wang

**Affiliations:** Department of General Surgery, China-Japan Friendship Hospital, Beijing 100029, China

## Abstract

**Objective:**

To investigate the efficacy and safety of the combination use of tegafur and apatinib as a first-line therapy strategy in advanced gastric cancer (GC).

**Methods:**

The present study included a total of 62 advanced GC patients. The patients were randomized into the combined group (treated with both tegafur and apatinib) and the control group (treated with only tegafur). Treatment efficacy, KPS score, nutrition condition, and progression-free survival time (PFS) were recorded.

**Results:**

Both the response and disease control rates were significantly higher in the combined group. The PFS time was remarkably higher and the KPS score was significantly reduced in the combined group after treatment. After treatment, both groups showed significantly increased nutrition risk, but the rates of patients with nutrition risk or innutrition were remarkably higher in the combined group. The ADR rates were also significantly higher in the combined group.

**Conclusion:**

The combination use could achieve good efficacy and prolong patients' PFS time; however, apatinib also reduced the patients' quality of life and enhanced the nutrition risk and adverse drug reactions.

## 1. Introduction

Gastric cancer (GC) is one of the most common cancers worldwide, with a high incidence of 640,000 cases for men and 350,000 cases for women in 2011 [1]. Since the early symptoms of GC are always slight and obscure, most GC patients develop to advanced stage upon diagnosis and thus have lost the best time for radical surgery [2, 3]. It is reported that the 5-year survival value is as low as 10% and the overall survival (OS) is limited to 1 year in the metastatic GC patients [4].

Chemotherapy is the key component in the treatment for advanced gastric patients [5]. However, patients have to suffer numerous adverse effects and great pain by conventional chemotherapeutic agents [6, 7]. In recent years, many new treatment methods are developed, such as checkpoint inhibition and target therapy, which are gradually applied in gastric cancer treatment [8–10]. Several drugs for the inhibition of tumor angiogenesis are developed, such as bevacizumab, sunitinib, sorafenib, and ramucirumab [11]. However, except for ramucirumab, most of these antiangiogenic agents failed to improve the patients' survival condition [12, 13]. Recently in 2014, apatinib, a kind of selective vascular endothelial growth factor receptor- (VEGFR-) 2 inhibitor, has been approved and showed good treatment efficacy for advanced gastric cancer [14]. Both preclinical and early clinical data demonstrated that apatinib had good *in vivo* efficacy in the treatment of gastric cancer [15]. Since apatinib is a new approved drug, it is usually used as a chemotherapeutic adjunctive drug in the treatment of gastric cancer. And whether it can be used as a first-line drug still lacks clinical evidences.

Tegafur is a widely used chemotherapy drug in many cancers, including gastric cancer. In an early research, it was reported that patients with early gastric cancer might show complete response for the treatment of tegafur [16]. Tegafur is also widely used in the treatment of advanced gastric cancer [17]. However, no study reported the combination use of apatinib and tegafur in the treatment of gastric cancer. In the present study, we aimed to investigate efficacy and safety of the combination use of tegafur and apatinib as a first-line therapy strategy in the treatment of advanced GC patients. This research might give more clinical evidences for apatinib in gastric cancer treatment.

## 2. Methods and Materials

### 2.1. Patients

The present single-blinded prospective randomized controlled study included a total of 69 patients with advanced gastric cancer who went to our hospital during January 2016 to August 2017. All patients were consecutively enrolled and all patients who met the inclusion criteria during this period were included. The enrollment of the patients was performed by 2 independent physicians who did not participate in the intervention process. The diagnosis of advanced gastric cancer was confirmed by histological analysis and all patients had gastric adenocarcinoma. Other inclusion criteria included the following: (1) the tumor-node-metastasis (TNM) stage for the patients were stage III~IV, (2) patients were primarily diagnosed as advanced gastric cancer or patients with recurrence after surgery, (3) the bone marrow reserve and liver function of the patients were basically normal, (4) the predicted survival time were ≥3 months with the Karnofsky performance status (KPS) score ≥ 60. Exclusion criteria included (1) patients who received other first-line chemotherapy before and (2) patients with other primary tumors. Written informed consent was obtained from all patients. The present study was approved by the Ethics Committee of China-Japan Friendship Hospital.

### 2.2. Treatment Strategy

After being enrolled in the study, the patients were randomized into two groups by a computer-generated list, (1) the combined group, in which patients received treatment of both tegafur and apatinib, and the control group, in which patients were treated with only tegafur. For the combined group, patients received apatinib (Jiangsu Hengrui Pharmaceutical Co., Ltd., China) with a dose of 500 mg/d and tegafur (Taiho Pharmaceutical Co., Ltd., Beijing, China) with doses of 40~60 mg twice a day. The control group only received treatment of tegafur. For treatment of apatinib, the patients were treated with the dose of 500 mg/d at first, and the dose could be gradually enhanced to 850 mg/d if the patients showed good tolerance. When patients showed severe side effects for apatinib, the dose could be reduced to 425~500 mg/d. For treatment of tegafur, the doses were according to the body surface area (BSA), 40 mg twice a day for BSA < 1.25 m^2^, 40 mg at morning and 60 mg at night for BSA within 1.25~1.50 m^2^, and 60 mg twice a day for BSA > 1.50 m^2^. Patients received treatment for 2 weeks and stopped for 1 week. The treatment for 21 d was considered a cycle, and both treatments lasted for 4 cycles. For both groups, blood routine, liver function, kidney function, and coagulation function were monitored and patients were regularly reviewed.

### 2.3. Data Collection and Measurement

The efficacy measurement was conducted for every treatment cycle. Treatment efficacy was evaluated according to the criteria of Response Evaluation Criteria in Solid Tumors Version 1.1 (RECIST 1.1) [18], including the complete response (CR), partial response (PR), stable disease (SD), and progressive disease (PD). The response rate was calculated as (CR + PR)/total × 100% and the disease control rate was calculated as (CR + PR + SD)/total × 100%. The adverse drug reaction (ADR) was defined according to WHOs criteria [19]. Patients quality of life was measured using the KPS score. The nutrition condition of the patients was evaluated according to the standard of Nutritional Risk Screening-2002 (NRS-2002), 0 for no nutrition risk, 1~2 for patients with nutrition risk, and ≥ 3 for innutrition. For survival analysis, the patients were followed up for 1 year or until death. The progression-free survival time (PFS) was defined as time from treatment to tumor progression.

### 2.4. Statistical Analysis

The measurement data was expressed as mean ± SD. Comparison between two groups of continuous data was performed using the Student's *t*-test. Chi-square test was used to compare the rates. Kaplan-Meier (K-M) curve was performed with the log-rank test for survival analysis. Values were considered to be statistically significant when *P* < 0.05. All calculations were made using SPSS 20.0.

## 3. Results

### 3.1. Patients' Characteristics

**Figure 1:**
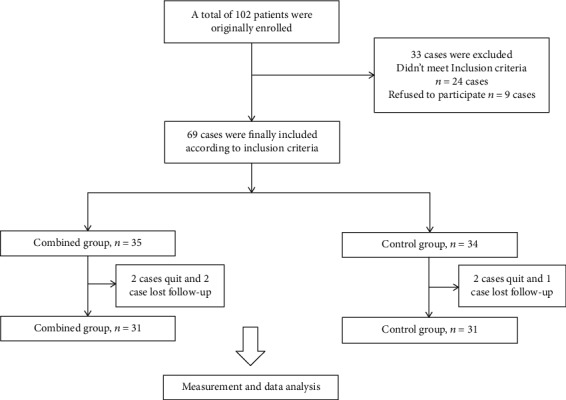
Flow chart.

The present study included a total of 69 patients with advanced gastric cancer who went to our hospital during January 2016 to August 2017. All patients were randomized into the combined group (*n* = 35) and the control group (*n* = 34). During the study period, 4 cases in the combined group and 3 cases in the control group quit the study or lost to follow-up. Patients' characteristics including age, gender, BMI, TNM stage, tumor differentiation, and KPS scores before treatment were recorded. As shown in Table 1, the mean age of the combined group was 65.0 ± 5.0, with male : female 19 : 12, and the mean age of the control group was 65.0 ± 6.0, with male : female 18 : 13. In all patients, 57 cases were TNM stage IV and 5 cases were TNM stage III. All the patients were followed up for 1 year or until death. No significant difference was observed between the two groups of patients. The flow chart is shown in Figure 1.

### 3.2. Treatment Efficacy and Survival Condition

**Figure 2:**
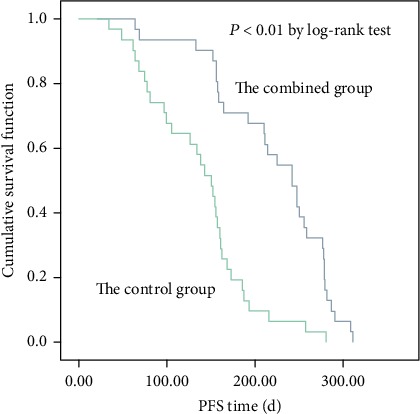
K-M curve for PFS time of the two groups of patients.

After 4 cycles of treatment, no case in both groups was observed as complete response. However, there are 2 (6.5%) cases in the combined group with PD, which significantly lower than that in the control group 10 (32.0%) (*P* < 0.05, Table 2). Meanwhile, both the response rate and the disease control rate were significantly higher than the control group (*P* < 0.05). For survival analysis, the median value of the PFS time for the patients was 8.1 month in the combined group, remarkably higher than that in the control group of 5.0 month, analyzed using K-M curve (*P* < 0.05, Figure 2). No patient was lost to follow-up during the study. These results suggested that the combined use of tegafur and apatinib might have better treatment efficacy and might prolong the PFS time.

### 3.3. Quality of Life and Nutrition Condition

To further investigate the effects of tegafur and apatinib on patients' condition, both KPS score and nutrition condition were collected before treatment and after 4 cycles of treatment. Results showed that the KPS score was significantly reduced in the combined group after treatment, while in the control group, the KPS score showed no significant difference before and after treatment (*P* < 0.05, Table 3). Similarly, the nutrition condition showed no significant difference between the two groups before the treatment. However, after treatment, both groups showed significantly increased nutrition risk (*P* < 0.05). And the rates of patients with nutrition risk or innutrition after treatment were remarkably higher in the combined group (*P* < 0.05), indicating that the combined use of tegafur and apatinib might reduce the patients' quality of life and enhance the nutrition risk.

### 3.4. Adverse Drug Reactions

Finally, we evaluated ADR during the study period. The ADR was measured according to the Common Terminology Criteria for Adverse Events Version 4.0. and was divided into 0~IV stages. As shown in Table 4, during the treatment, no stage IV ADR was found in both groups. The ADR rates for nausea, vomiting, hemoglobin decrease, proteinuria, and hypertension were significantly higher in the combined group than those in the control group (*P* < 0.05), suggesting that the combined use of tegafur and apatinib might enhance the ADR rate during treatment.

## 4. Discussion

Despite numerous studies and treatment development for gastric cancer, the treatment efficacy and prognosis of gastric cancer patients are still unsatisfied [20]. Since most gastric cancer patients are diagnosed as advanced stage, to prolong the patients' survival time and improve their quality of life become very important [21]. Apatinib, a novel antiangiogenesis drug approved by Chinese Food and Drug Administration, has been proven to be effective in the treatment of advanced gastric cancer patients. However, since apatinib is a new drug, reports of its application, efficacy, and side effects are still lacking. Generally, it is recommended that apatinib can be used for gastric cancer patients as a third-line agent who failed for the first-line and second-line chemotherapy [15]. However, in clinical experience, many doctors and scholars also found that it has the potential as a second- or first-line therapy drug. Zhang et al., in a recent research, showed that apatinib could be used as a second-line therapy for advanced gastric and gastroesophageal cancer with manageable toxicity [22]. In a more recent case report, it was also reported that the combination of apatinib with S-1 has the potential to be the first-line therapy for advanced gastric cancer [23]. In the present study, we demonstrated for the first time that the combination of tegafur and apatinib as first-line therapy could achieve good efficacy and prolong patients' progressive-free time in the treatment of advanced gastric cancer; however, apatinib also reduced the patients' quality of life and enhanced the nutrition risk and adverse drug reactions.

Application of apatinib in the treatment of gastric cancer has been already reported in several researches. In the phase III study of apatinib, it was reported that apatinib could significantly prolong the median overall survival and progression-free survival time for advanced GC patients [24]. In a case report, Zhu et al. demonstrated treatment of a 64-year-old Chinese female by apatinib; in this study, the patient's progression-free survival was 5 months after treatment of apatinib and the patient lived for a total of 22 months after tumor transfer [25]. However, apatinib is also reported to have several adverse drug reactions, such as hypertension, hand-and-foot syndrome, and proteinuria, [24]. In another case report, Li et al. reported a 55-year-old Chinese woman with advanced gastric cancer and observed gastrointestinal hemorrhage after treatment by apatinib for 19 d and finally died of septic shock [26]. In the present study, we used apatinib as the first-line therapy medicine and we also found that apatinib was able to enhance the response rate of the patients and prolong the progressive-free time. Meanwhile, side effects were also enhanced and the patients' quality of life was also influenced, as well as the nutrition risk. Thus, whether to use apatinib as a first-line drug still needs more studies to confirm.

Studies also indicate that apatinib can be used in the treatment of other cancers, such as non-small-cell lung cancer in a phase II trial [27]. In an *in vitro* research, authors found that apatinib could inhibit VEGF signaling and could promote cell apoptosis in intrahepatic cholangiocarcinoma [28]. It was also shown that apatinib could enhance the efficacy of conventional chemotherapeutical drugs in leukemia cells [29].

Tegafur is a widely used chemotherapy drug in a wide range of cancers, including gastric cancer [30], breast cancer [31], and colorectal cancer [32]. It was reported that tegafur could be used in metastatic gastric cancer; however, it might not enhance the patients' response to fluorouracil-based first-line chemotherapy [33]. In a phase II trial for advanced gastric cancer patients, the combination use of epirubicin, cisplatin, and oral tegafur plus leucovorin has a significant activity and tolerable toxicities in patients with gastric carcinoma [30]. In the present study, the application of tegafur also showed good treatment efficacy. Though the response rate and PSF time were not as well as the combination use of both apatinib and tegafur, single use of tegafur represented to be safer with fewer side effects and was better in patients' quality of life and nutrition condition.

In conclusion, we conducted a randomized controlled study to investigate efficacy and safety of the combination use of tegafur and apatinib as a first-line therapy in the treatment of advanced gastric cancer. Results showed that the combination use could achieve good efficacy and prolong patients' progressive-free time; however, apatinib also reduced the patients' quality of life and enhanced the nutrition risk and adverse drug reactions. Thus, to use apatinib as a first-line therapy medicine still needs more clinical evidences to give more data.

## Figures and Tables

**Table 1:** Patients' characteristics.

Variables	Combined group(*n* = 31)	Control group (*n* = 31)	*P* value
Age (year)	65.0 ± 5.0	65.0 ± 6.0	0.948
BMI (kg/m^2^)	20.6 ± 1.4	21.0 ± 1.4	0.268
Gender (male : female)	19 : 12	18 : 13	0.773
TNM stage, *n* (%)			0.460
III	3 (9.7)	2 (6.5)	
IV	28 (90.3)	29 (93.5)	
Tumor differentiation, *n* (%)			0.658
Poorly differentiated	28 (90.3)	27 (87.1)	
Moderately differentiated	3 (9.7)	4 (12.9)	
KPS score	81.4 ± 10.3	80.2 ± 12.0	0.674

**Table 2:** Treatment efficacy and survival condition for all patients.

Variables, *n* (%)	Combined group (*n* = 31)	Control group (*n* = 31)	*P*
CR	0 (0)	0 (0)	<0.001
PR	9 (29.0)	3 (9.7)	
SD	19 (61.3)	18 (58.1)	
PD	2 (6.5)	10 (32.0)	
Response rate	9 (29.0)	3 (9.7)	<0.001
Disease control rate	29 (93.5)	21 (67.7)	<0.001

Note: CR: complete response; PR: partial response; SD: stable disease; and PD: progressive disease.

**Table 3:** Quality of life and nutrition condition for all patients.

Variables	Combined group (*n* = 31)	Control group (*n* = 31)	*P*
KPS score			
Before treatment	81.4 ± 10.3	80.2 ± 12.0	0.674
After treatment	70.7 ± 13.4	80.1 ± 12.0	0.005
Nutrition condition, *n* (%)			
Before treatment			0.580
0	0 (0)	0 (0)	
1	15 (48.4)	14 (45.2)	
2	9 (29.0)	8 (25.8)	
≥3	7 (22.6)	9 (29.0)	
After treatment			0.009
0	0 (0)	0 (0)	
1	4 (12.9)	8 (25.8)	
2	11 (35.5)	13 (41.9)	
≥3	16 (51.6)	10 (32.2)	

**Table 4:** Adverse drug reactions for all patients.

Complication, *n* (%)	I	II	III	Total	I	II	III	Total	*P*
	Combined group (*n* = 31)				Control group (*n* = 31)				
Nausea	16	8	2	26 (83.9)	9	4	1	17 (54.8)	<0.001
Vomiting	11	7	1	19 (61.3)	5	1	0	9 (29.0)	<0.001
Hemoglobin decrease	8	8	2	18 (58.1)	9	2	0	11 (35.5)	0.002
Hypertension	8	7	1	16 (51.6)	2	1	0	3 (9.7)	<0.001
Leukopenia	13	9	3	25 (90.3)	15	7	2	24 (77.4)	0.013
Thrombocytopenia	4	1	0	5 (16.1)	5	1	0	6 (19.4)	0.541
Proteinuria	8	3	0	11 (35.4)	2	1	0	3 (9.7)	<0.001
Diarrhea	4	2	0	6 (19.3)	2	2	0	4 (12.9)	0.218
Hand-and-foot syndrome	6	3	2	11 (35.4)	5	4	1	10 (32.2)	0.765
Fatigue	5	4	0	9 (29.0)	4	3	0	7 (22.6)	0.301

## Data Availability

The data used to support the findings of this study are included within the article.
